# Ridge-furrow mulched with plastic film improves the anti-oxidative defence system and photosynthesis in leaves of winter wheat under deficit irrigation

**DOI:** 10.1371/journal.pone.0200277

**Published:** 2018-07-11

**Authors:** Shahzad Ali, Yueyue Xu, Qianmin Jia, Irshad Ahmad, Xiangcheng Ma, Malak Henchiri, Xiaolong Ren, Peng Zhang, Tie Cai, Jiahua Zhang, Zhikuan Jia

**Affiliations:** 1 College of Agronomy, Northwest A&F University, Yangling, Shaanxi, China; 2 School of Computer Science and Technology, Remote Sensing and Climate Change, Qingdao University, Shandong, China; Huazhong Agriculture University, CHINA

## Abstract

In semi-arid areas of China, the ridge-furrow mulched with plastic film (RF) cultivation system is a common water-saving agricultural technique where the shortage of water resources has become a serious problem. Therefore, we aimed to explore whether this cultivation is actually an improvement over the traditional flat planting (TF) method while testing two deficit irrigation (150, 75 mm) levels to grow winter wheat. Furthermore, we examined the responses of the anti-oxidative defence system and photosynthetic capacity of winter wheat flag leaves under three simulated rainfall (275, 200 and 125 mm) conditions. The results showed that the RF system with 150 mm deficit irrigation and 200 mm simulated rainfall condition (RF2_150_) treatment raised soil water content (%) at the jointing and flowering stages and achieved higher net photosynthesis rates (Pn) in flag leaves. Furthermore, such improvements were due to the reduction of malondialdehyde (MDA) content and oxidative damage during different growth stages of winter wheat. The RF2_150_ treatment significantly increased the activities of superoxide dismutase (SOD); peroxidise (POD), catalase (CAT) and ascorbate peroxidase (APX) and the content of soluble protein (SP) during different growth stages of winter wheat. Furthermore, RF2_150_ treatment attained the highest value at the flowering stage, while also exhibiting significant declines in contents of proline, MDA, H_2_O_2_ and O_2_ in flag leaves. The higher free H_2_O_2_ and O_2_ scavenging capacity and better anti-oxidative enzyme activities under the RF2_150_ treatment were due to the lower level of lipid peroxidation, which effectively protected the photosynthetic machinery. The net photosynthetic rate of flag leaves was positively correlated with SOD, POD, CAT, APX and SP activities, and negatively correlated with proline, MDA, H_2_O_2_ and O_2_ contents. We concluded that the RF2_150_ treatment was the better water-saving management strategy because it significantly delayed flag leaf senescence and caused the increases in SWC, WUE, Pn, antioxidant enzyme activities and grain yield of winter wheat grown in semi-arid regions of China.

## Introduction

In semi-arid regions of China, water shortages due to inadequate and unpredictable rainfall have restricted plant growth and development more so than any other environmental factor [1]. Drought stress decreases the photosynthetic capacity of a crop to different degrees during its various growth stages [2]. During the critical growth stages of crops, water shortages induce leaf senescence; decrease photosynthetic capacity and cause oxidative damage [3]. Furthermore, [4] estimated that in 2050, drought stress will cause serious harm to crop growth in more than 50% of arable lands. Despite the low amounts of precipitation in semi-arid regions, it is important to take advantage of the light rainfall events to increase soil water storage [5]. The primary agricultural practice in these semi-arid areas used to sustain crop growth and yield is through the combinatorial use of light rainfall events and deficit irrigation [6, 7]. Plastic film mulching with the ridge-furrow rainfall harvesting (RFRH) system is an effective technique employed to collect water from light precipitation events to improve rainwater use efficiency [8, 9]. Therefore, in this present study, we investigated the interactive effects of cultivation models with deficit irrigation strategies to improve the anti-oxidative defence system and photosynthetic capacity of winter wheat flag leaves under simulated rainfall conditions.

Many physiological changes occur in crops under drought stress. Drought stress can negatively affect grain filling and photosynthetic rates in crops by reducing low soluble protein (SP) content in flag leaves and thus, plant sucrose content [10]. Water stress induces stomatal closure, which reduces the diffusion of CO_2_ from the atmosphere into the cell and consequently, decreases photosynthetic activity [11]. During drought conditions, supplemental irrigation promotes plant tissue development, leading to significant increases in photosynthesis and SP content of flag leaves [12]. Drought stress cause to decrease the Pn and total Chl ab, at the grain filling stage wheat leaves start to senesce and the photosynthetic machine quickly disassembles [13]. This leaves senesce linked with decline in photosynthetic capacity of flag leaves [14].

Drought stress promotes the production of reactive oxygen species (ROS) such as H_2_O_2_ and O_2_^-^, which leads to chlorophyll damage and a decrease in the chlorophyll stability index [15]. Plant water stress is often linked to increased oxidative stress owing to enhanced accumulation of ROS [16, 17]. Normal plant metabolism can be destroyed by ROS through oxidative damage to lipids, proteins and nucleic acids. Therefore, oxidative damage negatively affects plant performance, Pn and components of chlorophyll [18]. Reactive oxygen species can cause damage of cell membrane and produce malondialdehyde (MDA). The content of MDA in wheat significantly increased and reached maximal levels at the mature stage while under water stress compared to that under conditions without water stress [19]. However, crops have developed advanced anti-oxidant defence systems using enzymes, such as superoxide dismutase (SOD), peroxidise (POD), catalase (CAT) and ascorbate peroxidase (APX), to reduce H_2_O_2_ and O_2_^-^ production [20]. Thus, attaining higher activity rates of SOD, POD, CAT and APX and low MDA, H_2_O_2_ and O_2_^-^ production in flag leaves is a key strategy to improve the photosynthetic capacity and chlorophyll content of plants under drought conditions [21]. Amino acid proline is known to be the first responding enzyme in plants exposed to water stress and plays sufficient roles in plant stress tolerance [22, 23]. Proline acts as an osmolyte and promotes plant damage repair by increasing anti-oxidant activity during drought conditions [24]. Thus, strategies to protect the flag leaves from oxidative damage and delay the senescence process are essential to improving the anti-oxidative defence system [25]. However, to the best of our knowledge, no studies have been carried out to understand the plant response to drought-induced oxidative stress and photosynthetic capacity of winter wheat flag leaves under field conditions during complete life cycle of wheat. The purposes of the present study were to clarify the responses of two cultivation models under deficit irrigation and effects of different simulated rainfall conditions in water-stressed plants based on the differentiation of its improving soil water content from physiological anti-drought function. The interaction effects of cultivation models, deficit irrigation and simulated rainfall conditions in relation to production, antioxidant metabolism and detoxifying system of ROS are also examined.

## Materials and methods

### Study site description

The field study was performed during 2015–2017 at the Northwest A&F University, Shaanxi Province, China (34°20'N, 108°24'E). The experimental site was 466.7 m above sea level, annual mean temperature is 12.9°C, and the annual evaporation rate was 1753 mm. The total duration of daylight was 2196 hours per year, with a frost-free period of 220 days per year. The mean soil bulk density was 1.37 g cm^−3^. The averages of two years of available NPK data were 39.4 mg kg^-1^, 7.98 mg kg^-1^ and 99.94 mg kg^-1^. At the 0–20 cm soil layer, the soil organic matter was 10.88 g kg^−1^ and the pH was 7.80.

### Experimental design and treatments

The trial was conducted in large-sized waterproof sheds. The internal shed dimensions were 32 m (length) ×15 m (width) × 3 m (height). The sheds had a transparent plastic-covered roof and four open sides. The mobile sheds were used to manage natural precipitation. In this research, we adopted the use of a rainfall simulator (RS) to supply the crop water requirements, and no natural precipitation occurred during the winter wheat growing season. The RS was used according to methods in a previous study [26]. In the precipitation simulation, three total seasonal precipitations, 125, 200, and 275 mm, corresponded to light, moderate, and heavy rainfall levels. This precipitation level partitioning was derived from the spatial and temporal characteristics of the precipitation distribution in the semi-arid regions of China over the past 50 years. A detailed description of the precipitation determination is shown in Table 1. In this simulation research, the partition of the application volume in pulsed precipitation events was not completely realistic under field conditions but was reasonably close.

**Table 1:** Partition of rainfall simulation during winter wheat-growing seasons.

Growth stages	Rainfall events	Rainfall duration	Daily rainfall distribution (mm)		
			125 mm	200 mm	275 mm
Seedling	2	28–29 October	25	32	40
		24–25 November	13	22	30
Wintering	2	18–19 December	4	5	4
		22–23 January	3	5	6
Green	1	26–27 February	5	10	12
Jointing	1	20–21 March	15	24	43
Grain filling	3	9–10 April	15	30	25
		26–27 April	15	22	25
		10–11 May	15	25	45
Maturing	1	23–24 May	15	25	45

In 2015–17, we conducted field research trial to explore the potential role of two cultivation models: (1) the ridge-furrow rainfall harvesting (RF); and (2) traditional flat planting (TF), under two deficit irrigation (150, 75 mm) levels and three simulated rainfall (1: 275, 2: 200, 3: 125 mm) levels in a randomized complete block design (RCBD) with three replicates. Using a precise water meter, half of the deficit irrigation was supplied on December 12, 2015 and December 15, 2016 (before the re-wintering stage) and the other half was supplied on March 28, 2016 and March 25, 2017 at the jointing stage. The deficit irrigation volumes for 150 and 75 mm were measured according to the irrigation area. The irrigation area for the TF cultivation treatment was 6.3 m^2^ (2.0 m × 3.15 m) and the irrigation volume was 0.95 and 0.47 m^3^ under 150 and 75 mm, respectively. The irrigation area under the RF technique of the two furrows was 3.78 m^2^ (1.2 m × 3.15 m) and the irrigation volumes of the two furrows were 0.57 and 0.28 m^3^. The RF technique used a ridge height of 15 cm with a ratio of furrow to ridge widths of 60:40 cm. Four rows of wheat were sown in furrows. Weeds were controlled manually during each growing season of the winter wheat crop. Wheat cultivar (Xinong 979) was sown at the rate of 2.25 × 10^6^ seeds ha^-1^. The seeds were planted with an inter-row space of 20 cm on October 15 in 2015 and on October 10 in 2016. Wheat was hand harvested on June 2 in 2016 and on May 27 in 2017. Nitrogen (urea) at 225 kg ha^-1^ and phosphorus (diammonium phosphate) at75 kg ha^-1^ were applied at the time of planting.

### Data collection

#### Soil water content

The soil water content (SWC) was calculated at the sowing time, jointing stage, flowering stage, grain filling stage and maturity stage during 2015–2016 and 2016–2017 study year. Moisture contents of the 0–100 cm soil layers at 10 cm intervals were recorded using a TDR meter (Time-Domain Reflectometry, Germany).

The seasonal evapotranspiration rate was calculated using the following Eq (1) [26]:

ET=P+I+ΔW (1)

where P (mm) is the precipitation; I (mm) is the irrigation; ΔW (mm) is the soil moisture content for the 0–200 cm soil depths between planting time and maturity stage, or between the growth stages.

Water use efficiency (WUE, kg ha^−1^ mm^−1^) was calculated using the following Eq (2) [26]:

WUE=Y/ET (2)

where Y is the grain yield (kg ha^-1^) which was measured at maturity in the central four rows of each plot, including the combined area of the ridges and furrows, and ET is the total evapotranspiration (mm) over the growing season.

#### Photosynthetic and estimation of photosynthetic pigments

The net photosynthetic rate (Pn) was measured using a LI-Cor LI-6400XT portable photosynthesis system (LI-6400XT, LI-Cor, Lincoln, NE, USA). Measurements from the fully expanded flag leaves were taken on sunny days between 9:00 and 11:00 a.m. The CO_2_ concentration in the leaf chamber was set at 380 μmol mol^-1^ and the photosynthetic active radiation was set at 1100 μmol m^-2^ s^-1^. At jointing, flowering and grain filling stage, 9 leaves from five individual plants in each of the three replicates of each treatment were analyzed.

#### Enzyme extracts preparation

0.5 g of flag leaves with removed midrib was homogenized 5 mL of 50 mmol L^-1^ phosphate buffer (pH 7.8), 0.1 mM EDTA-Na_2_ and 1% insoluble PVP. The homogenate was centrifuged for 10 minutes at 15,000 x g at 4 ^0^C. After centrifuged the upper supernatant was taken and used for enzyme assay.

#### Assays of antioxidant enzyme activities

Total superoxide dismutase (SOD) activity was analyzed at 560 nm according to the technique of [27, 28, 29]. SOD activity was expressed as U g^-1^ FW h^-1^. POD activity was calculated with guaiacol at 470 nm according to the technique of [30]. POD activity was expressed as U g^-1^ FW min^-1^. Assayed of CAT activity was according to the method of [9]. CAT activity was expressed as U g^-1^ FW min^-1^. Analyzed of MDA content was according to the technique of [31]. MDA content was expressed as μmol g^-1^ FW. According to the Coomassie brilliant blue (G250) method described by [32] soluble protein concentration of flag leaves was measured. Soluble protein content was expressed as mg g^-1^ FW.

#### Measurement of O_2_^-^ and H_2_O_2_

O_2_^-^ content was determined by the modification of the method of [33] described by [34]. 200 mg of powered fresh samples were homogenized with 1,000 μL of 65 mM phosphate buffer (pH 7.8) and centrifuged at 10,000 rpm for 10 min. 100 μL of the clear supernatants were mixed with 75 μL of phosphate buffer (pH 7.8) and 25 μL of 10 mM hydroxylamine hydrochloride, and incubated at room temperature for 20 min. After derivatization with 400 μL of 17 mM La-sulfanilic acid and 400 ml of 7 mM _L_-1-α-naphthylamine, O_2_^-^ content was analyzed at 530 nm. Superoxide radicals production rate was expressed μmol g^-1^ FW.

To determine H_2_O_2_ concentrations, 200 mg of powered fresh samples were homogenized with 400 μl of acetone and centrifuged at 3,000 rpm for 10 min. H_2_O_2_ content in the supernatants was analyzed at 415 nm [35, 34]. H_2_O_2_ content was expressed as μmol g^-1^ FW.

#### APX activity and proline contents

APX activity was assayed by monitoring the rate of reduced ascorbate (AsA) oxidation at 290 nm and was calculated using an extinction coefficient (ε) of 2.8 mmol L^–1^ cm^–1^, according to [36]. Proline content in the leaves was analyzed at 520 nm according to the technique of [37]. The proline content (μmol g^-1^ FW) in the samples was calculated using the standard curve.

#### Statistical analysis

The data were analysed using SPSS 18.0, and data obtained from each sampling event were analysed separately. Multiple comparisons were tested with Duncan’s new multiple range test. Mean values were evaluated through (LSD 0.05) multiple comparison tests, if the F tests were significant.

## Results

### Soil water content (%), net photosynthetic rate (Pn) and soluble protein (SP) content

**Fig 1:**
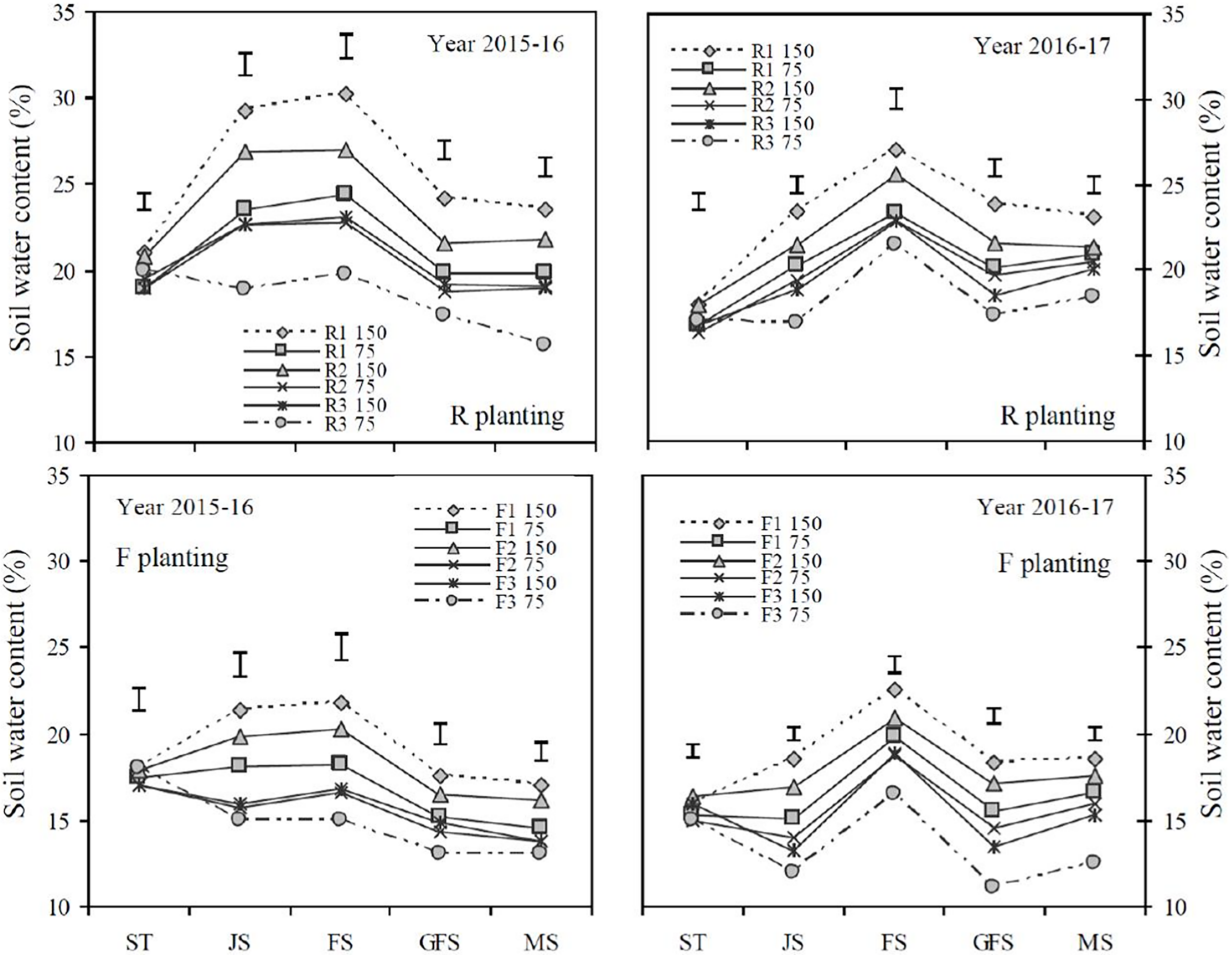
Effects of different cultivation models and deficit irrigation on average soil water content (%) at the depth of 0–100 cm soil layers under simulated rainfall conditions at different growth stages of winter wheat during 2015–2017. Note: R_150_: ridges covered with plastic film mulch and 150 mm deficit irrigation; R_75_: ridges covered with plastic film mulch and 75 mm deficit irrigation; F_150_: traditional flat cultivation and 150 mm deficit irrigation; F_75_: traditional flat cultivation and 75 mm deficit irrigation. Three different simulated rainfall concentrations 275 mm, 200 mm and 125 mm were used. ST: sowing time; JS: jointing stage; FS: flowering stage; GFS: grain filling stage; MS: maturing stage. Bars represent the LSD at p = 0.05 (n = 3).

The R system had significantly greater soil water content (SWC) at soil depths of 0–100 cm during various growth stages of wheat under various simulated precipitation and deficit irrigation levels than that of the F system (P < 0.05, Fig 1). There were non-significant differences in SWC at sowing time (ST) among all the treatments during both study years. Samples from the R1_150_ treatment had significantly higher SWC than that of all of the other treatments at each growth stage (with the exception of the R2_150_ treatment). The SWC was significantly greater under the R system as the precipitation and irrigation levels increased more than that of F method. During the two years of study, the R system significantly increased SWC by 30.4% more than that of F system. The SWC significantly increased from the jointing to the flowering stages, while SWC showed a significantly decreasing trend from the flowering to the grain filling and harvesting stages among all the treatments during both study years. The SWC significantly increased by 13.4% more in the R2_150_ treatment than that of R3_150_ treatment.

**Fig 2:**
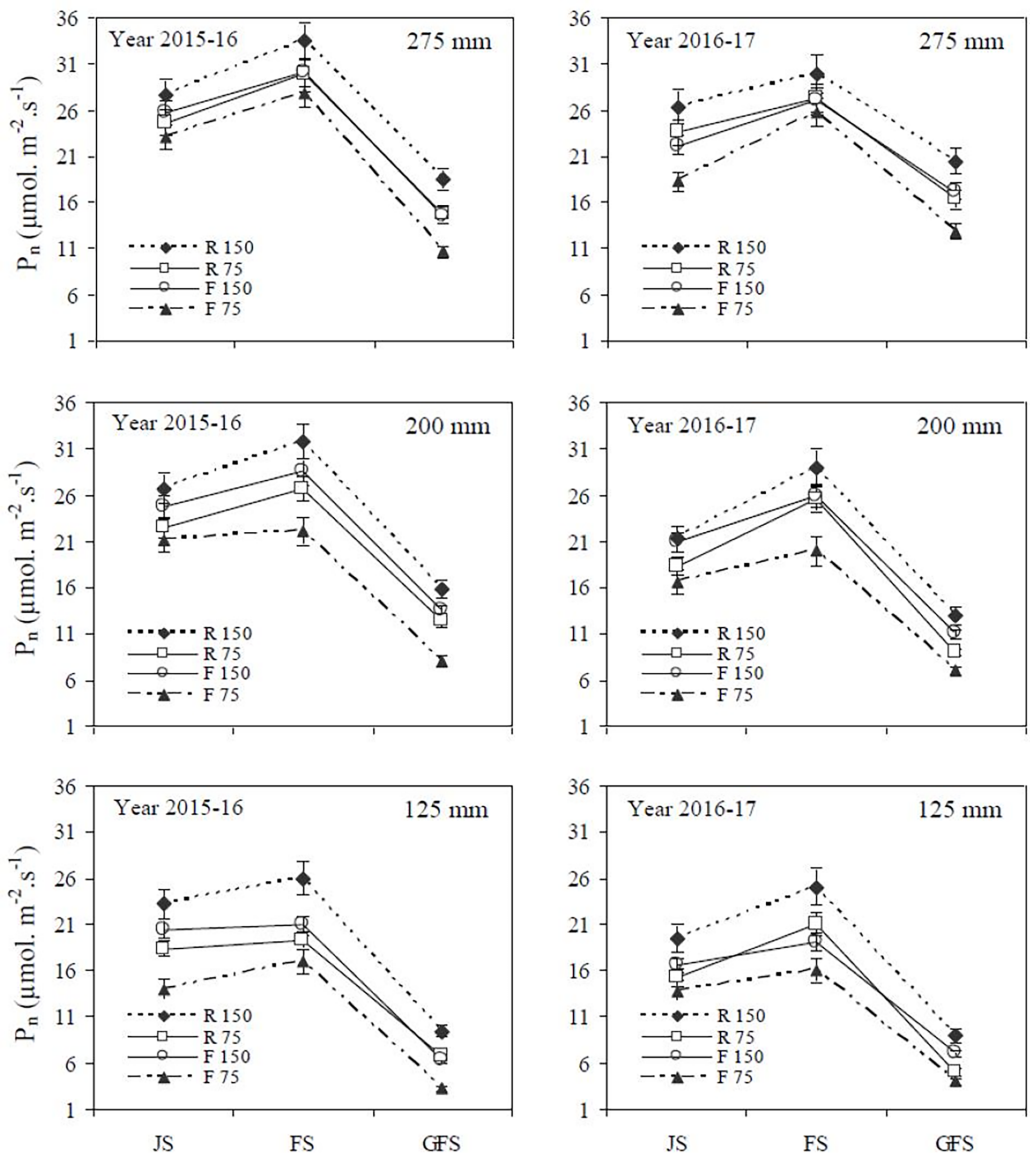
Effects of different cultivation models and deficit irrigation on net photosynthesis rate (Pn) of winter wheat flag leaves under simulated rainfall conditions during 2015–2017 growing seasons. Note: R_150_: ridges covered with plastic film mulch and 150 mm deficit irrigation; R_75_: ridges covered with plastic film mulch and 75 mm deficit irrigation; F_150_: traditional flat cultivation and 150 mm deficit irrigation; F_75_: traditional flat cultivation and 75 mm deficit irrigation. Three different simulated rainfall concentrations 275 mm, 200 mm and 125 mm were used. JS: jointing stage; FS: flowering stage; GFS: grain filling stage. Bars represent the LSD at p = 0.05 (n = 3).

**Fig 3:**
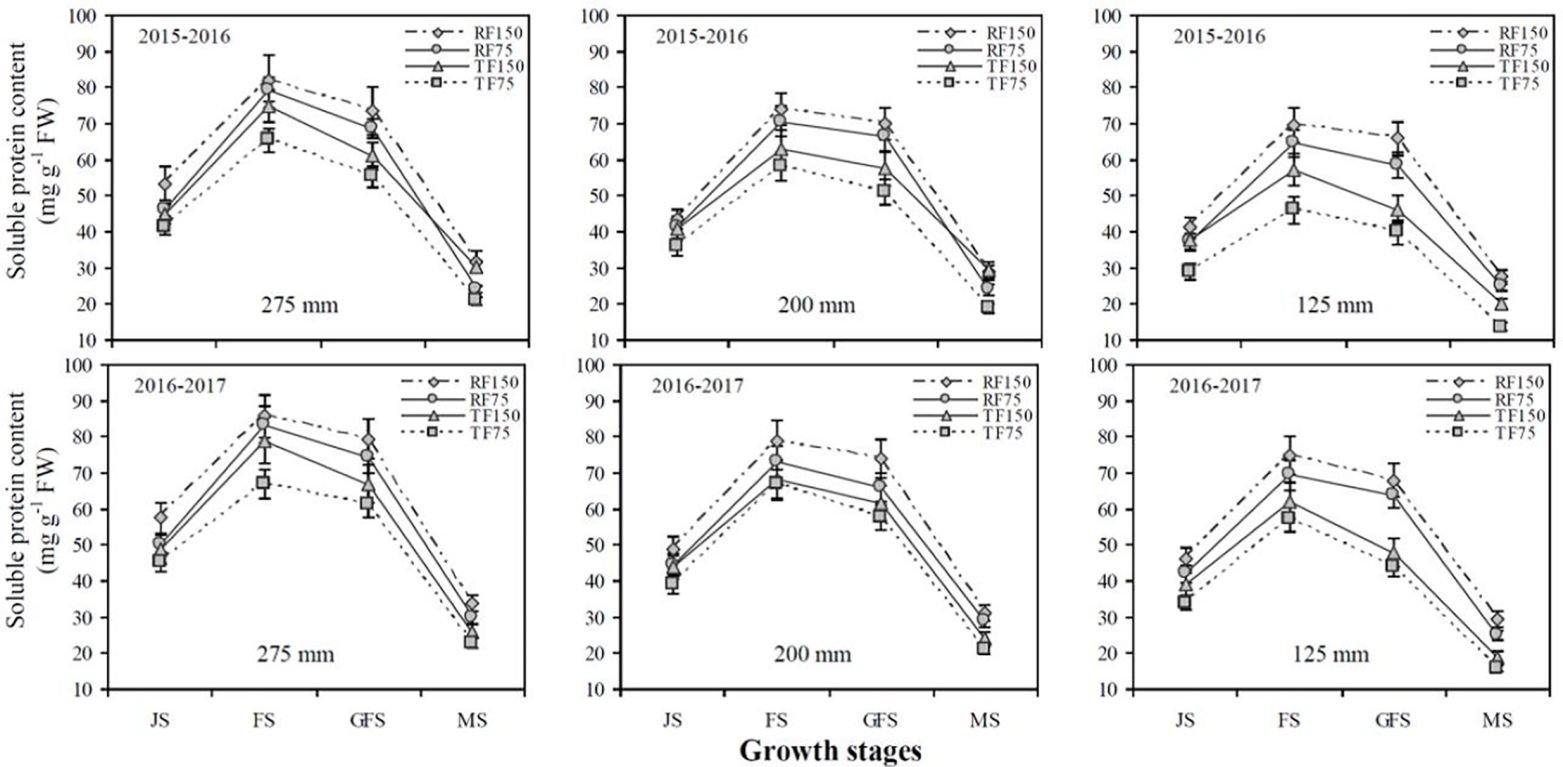
Effects of different cultivation models and deficit irrigation on soluble protein content (SP) content of winter wheat flag leaves under simulated rainfall conditions during 2015–2017 growing seasons. Note: RF1: ridges covered with plastic film mulch and 275 mm simulated rainfall; RF2: ridges covered with plastic film mulch and 200 mm simulated rainfall; RF3: ridges covered with plastic film mulch and 125 mm simulated rainfall; TF1: traditional flat planting and 275 mm simulated rainfall; TF2: traditional flat planting and 200 mm simulated rainfall; TF3: traditional flat planting and 125 mm simulated rainfall; 150: 150 mm deficit irrigation; 75: 75 mm deficit irrigation. Bars represent the LSD at p = 0.05 (n = 3). Abbreviations are: JS: jointing stage, FS: flowering stage, GFS: grain filling stage, MS: maturity stage.

The Pn and SP content of winter wheat flag leaves significantly increased with increasing precipitation concentration and irrigation levels under both cultivation methods at different growth stages. The SP content was closely correlated to Pn and significantly increased the photosynthetic rates of flag leaves. Greater SP content in flag leaves as a result of improve soil water content can sustain a higher Pn (Figs 2 and 3). The Pn and SP content of wheat flag leaves were significantly higher from the jointing to flowering stages, whereas, they significantly declined from the flowering to grain filling stages in the two years of the same treatments (Figs 2 and 3). The mean Pn and SP content under the RF1_150_, RF2_150_ and RF3_150_ treatments were significantly greater by (14.5% and 13.5%), (10.4% and 12.7%) and (25.8% and 23.7%), respectively than those in the TF1_150_, TF2_150_ and TF3_150_ treatments. In the two years of study, Pn and SP content under the RF1_150_ and RF2_150_ treatments were significantly higher than those in the TF1_150_ and TF2_150_ treatments at the jointing, flowering and grain filling stages. However, when precipitation increased from 200 to 275 mm, there were no significant differences recorded in Pn and SP content under both cultivation methods at different growth stages. The RF system had significantly affected the Pn and SP content of flag leaves at each simulated precipitation level and deficit irrigation concentration during the later growth stages of winter wheat, whereas, the TF system did not.

### Superoxide dismutase (SOD), peroxidise (POD), catalase (CAT) and ascorbate peroxidase (APX) activities

**Fig 4:**
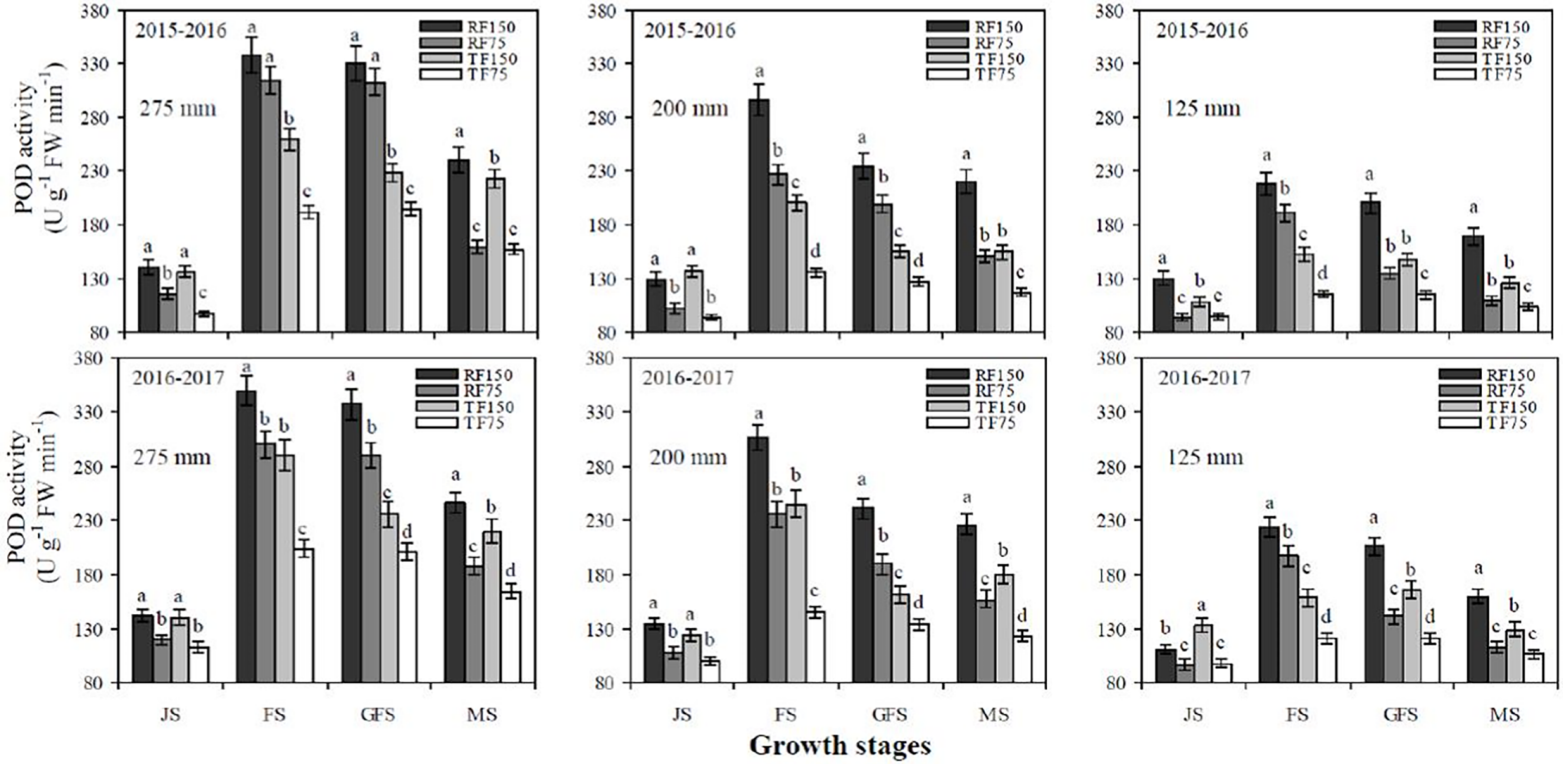
Effects of different cultivation models and deficit irrigation on peroxidase (POD) activity of winter wheat flag leaves under simulated rainfall conditions during 2015–2017 growing seasons. Note: RF1: ridges covered with plastic film mulch and 275 mm simulated rainfall; RF2: ridges covered with plastic film mulch and 200 mm simulated rainfall; RF3: ridges covered with plastic film mulch and 125 mm simulated rainfall; TF1: traditional flat planting and 275 mm simulated rainfall; TF2: traditional flat planting and 200 mm simulated rainfall; TF3: traditional flat planting and 125 mm simulated rainfall; 150: 150 mm deficit irrigation; 75: 75 mm deficit irrigation. Bars represent the LSD at p = 0.05 (n = 3). Abbreviations are: JS: jointing stage, FS: flowering stage, GFS: grain filling stage, MS: maturity stage.

**Fig 5:**
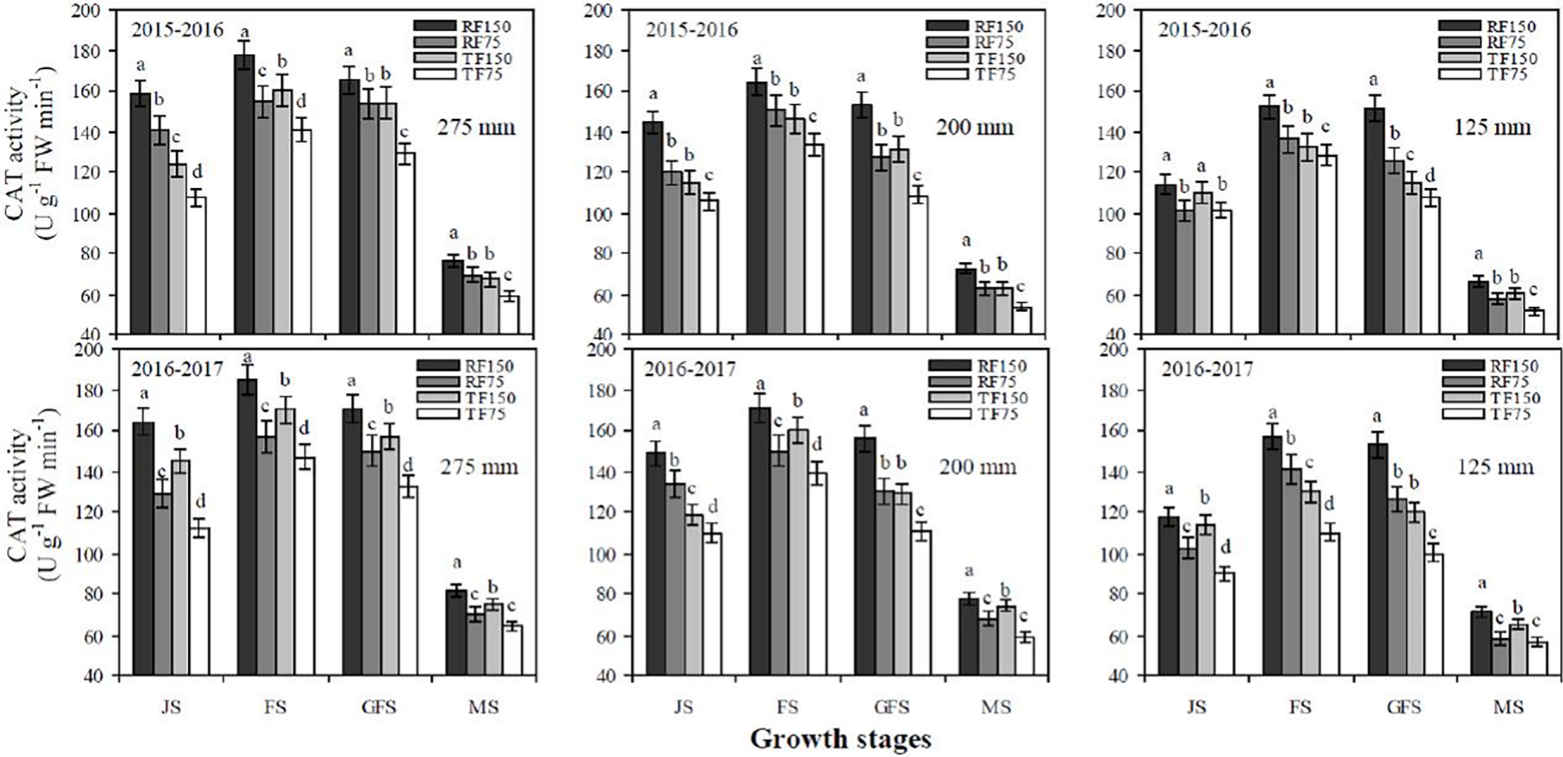
Effects of different cultivation models and deficit irrigation on catalase (CAT) activity of winter wheat flag leaves under simulated rainfall conditions during 2015–2017 growing seasons. Note: RF1: ridges covered with plastic film mulch and 275 mm simulated rainfall; RF2: ridges covered with plastic film mulch and 200 mm simulated rainfall; RF3: ridges covered with plastic film mulch and 125 mm simulated rainfall; TF1: traditional flat planting and 275 mm simulated rainfall; TF2: traditional flat planting and 200 mm simulated rainfall; TF3: traditional flat planting and 125 mm simulated rainfall; 150: 150 mm deficit irrigation; 75: 75 mm deficit irrigation. Bars represent the LSD at p = 0.05 (n = 3). Abbreviations are: JS: jointing stage, FS: flowering stage, GFS: grain filling stage, MS: maturity stage.

Table 2, Fig 4 and Fig 5 show that SOD, POD and CAT activities of flag leaves significantly increased with increasing precipitation and irrigation levels at the jointing, flowering, grain filling and maturity stages under both cultivation methods. The maximum SOD, POD and CAT activities of flag leaves were recorded under the RF1_150_ treatment at the flowering stage but had no significant difference with that of the grain filling stage, after which the SOD, POD and CAT activities of flag leaves quickly declined at the maturity stage. In addition, the difference between the RF1_150_ and RF2_150_ treatments was not significant at all growth stages of winter wheat. For both cultivation methods, SOD, POD and CAT activities of flag leaves significantly increased from the jointing to flowering stages and sharply declined from the grain filling to maturity stages at each deficit irrigation and simulated rainfall level. The RF cultivation method had significantly greater effects on the SOD, POD and CAT activities of flag leaves during the different growth stages than in the TF cultivation method. The two years of data of the four different growth stages revealed that the mean values of SOD, POD and CAT activities of flag leaves under the RF1_150_, RF2_150_ and RF3_150_ treatments were significantly greater by 5.6%, 23.4% and 14.4%), (12.9%, 35.3% and 18.5%) and (18.1%, 29.2% and 15.9%), respectively than those in the TF1_150_, TF2_150_ and TF3_150_ treatments. There were non-significant effects on SOD, POD and CAT activities of flag leaves when plants were treated with simulated rainfall of 275 mm or 200 mm levels at the jointing, flowering, grain filling and maturity stages.

**Table 2:** Superoxide dismutase (SOD) activity (U g^-1^ FW h^-1^) in winter wheat leaves of different treatments^a^ during 2015–2016 and 2016–2017^b^.

Treatments	Deficit irrigation	SOD activity (U g^-1^ FW h^-1^)							
		2015–2016				2016–2017			
		JS	FS	GFS	MS	JS	FS	GFS	MS
RF1	150	614a	805a	786a	543a	637a	815a	801a	555a
RF1	75	591b	766c	708c	442c	610c	777b	725c	452d
RF2	150	599b	780b	747b	523b	633a	813a	766b	533b
RF2	75	587c	752c	681d	420d	609c	762c	700c	429e
RF3	150	591b	740d	716c	413d	622b	773b	754b	500c
RF3	75	583c	688e	651e	258e	604c	698d	670d	358f
	**mean**	**594A**	**755A**	**715A**	**433A**	**619A**	**773A**	**736A**	**471A**
TF1	150	584a	773a	762a	465a	617a	790a	793a	479a
TF1	75	565b	734b	675b	385c	595b	750b	700b	399c
TF2	150	553b	688c	647c	418b	620a	714c	706b	430b
TF2	75	461d	656d	634c	366d	490e	670d	659c	199e
TF3	150	519c	678c	544d	349e	559c	700c	609d	366d
TF3	75	511c	650d	520e	145d	540d	668d	544e	169f
	**mean**	**532B**	**697B**	**630B**	**355B**	**570B**	**715B**	**669B**	**340B**
Analysis of variance	CM	*	*	*	*	*	*	*	*
	R	*	*	*	*	*	*	*	*
	DI	*	*	*	*	*	*	*	*
	CM x R	ns	ns	*	ns	ns	ns	ns	ns
	CM x DI	ns	ns	*	ns	ns	ns	*	*
	R x DI	ns	ns	ns	ns	ns	ns	ns	ns
	CM x R x DI	ns	ns	ns	ns	ns	ns	ns	ns

^a^ RF1: ridges covered with plastic film mulch and 275 mm simulated rainfall; RF2: ridges covered with plastic film mulch and 200 mm simulated rainfall; RF3: ridges covered with plastic film mulch and 125 mm simulated rainfall; TF1: traditional flat cultivation and 275 mm simulated rainfall; TF2: traditional flat cultivation and 200 mm simulated rainfall; TF3: traditional flat cultivation and 125 mm simulated rainfall; _150_: 150 mm deficit irrigation; _75_: 75 mm deficit irrigation. Abbreviations are: JS: jointing stage, FS: flowering stage, GFS: grain filling stage, MS: maturity stage.

^b^ Values are given as means, lowercase and uppercase letters indicate significant differences at P ≤0.05 levels in the same line (LSD test) comparisons among different treatments and between two cultivation models, respectively. ns denotes no significant different

*significant different at the 0.05 probability level; CM is the cultivation models, R is the rainfall simulator, DI is the deficit irrigation.

Under the RF system, the APX activity of flag leaves was significantly greater at each deficit irrigation and simulated rainfall level than that of the TF system (Table 3). However, data of the two years from the four different growth stages of winter wheat revealed that the RF1_150_ and RF2_150_ treatments had significantly greater mean APX activity in flag leaves, by 29.3% and 11.3% respectively, than that of the RF3_150_ treatment. Under the RF1_75_ and RF2_75_ treatments, APX activity of winter wheat flag leaves were significantly higher by 30.1% and 6.6%, respectively, than that of the RF3_75_ treatment. Under both cultivation methods, the APX activity of flag leaves significantly increased from jointing to flowering stages, while rapidly increased from flowering to maturity stages at each irrigation and simulated rainfall concentrations.

**Table 3:** Ascorbate peroxidase (APX) activity (nkat mg^–1^ protein) in winter wheat leaves of different treatments^a^ during 2015–2016 and 2016–2017^b^.

Treatments	Deficit irrigation	APX activity (nkat mg^–1^ protein)							
		2015–2016				2016–2017			
		JS	FS	GFS	MS	JS	FS	GFS	MS
RF1	150	76.2a	85.6a	112.0a	121.0a	80.0a	97.4a	116.8a	122.9a
RF1	75	70.7b	79.5b	97.0b	106.0b	74.2b	84.8b	99.9b	104.6b
RF2	150	75.5a	82.0a	87.1c	96.1c	79.6a	90.0a	94.2b	102.2b
RF2	75	61.4c	68.8d	72.8d	81.8d	63.9c	73.6c	78.0d	87.7c
RF3	150	72.8b	73.3c	75.3d	84.3d	76.8a	79.7b	83.4c	92.5c
RF3	75	59.4c	61.4e	68.0d	77.0d	61.9c	68.1c	74.9d	83.5d
	**mean**	**69.3A**	**75.1A**	**85.4A**	**94.4A**	**72.7A**	**82.3A**	**91.2A**	**98.9A**
TF1	150	72.6a	78.5a	85.1a	91.1a	74.8a	82.9a	91.7a	97.7a
TF1	75	70.3a	70.6b	79.4b	85.4b	73.5a	74.6b	85.4b	91.4b
TF2	150	63.7b	75.0a	79.4b	85.4b	66.9b	77.9a	85.6b	91.6b
TF2	75	61.4b	63.4c	64.8c	70.8c	64.6b	67.4c	70.1c	76.1c
TF3	150	61.0b	65.2c	70.8c	76.8c	64.3b	67.6c	74.8c	79.8c
TF3	75	51.8c	57.8d	60.1d	66.1d	53.7c	62.0d	65.5d	71.5d
	**mean**	**63.5B**	**68.4B**	**73.3B**	**79.3B**	**66.3B**	**72.1B**	**78.9B**	**84.7B**
Analysis of variance	CM	*	*	*	*	*	*	*	*
	R	*	*	*	*	*	*	*	*
	DI	ns	ns	*	ns	ns	*	*	ns
	CM x R	*	ns	*	*	ns	ns	*	*
	CM x DI	ns	ns	ns	ns	*	ns	ns	ns
	R x DI	ns	ns	ns	ns	ns	ns	ns	ns
	CM x R x DI	ns	ns	ns	ns	ns	ns	ns	ns

^a^ RF1: ridges covered with plastic film mulch and 275 mm simulated rainfall; RF2: ridges covered with plastic film mulch and 200 mm simulated rainfall; RF3: ridges covered with plastic film mulch and 125 mm simulated rainfall; TF1: traditional flat cultivation and 275 mm simulated rainfall; TF2: traditional flat cultivation and 200 mm simulated rainfall; TF3: traditional flat cultivation and 125 mm simulated rainfall; _150_: 150 mm deficit irrigation; _75_: 75 mm deficit irrigation. Abbreviations are: JS: jointing stage, FS: flowering stage, GFS: grain filling stage, MS: maturity stage.

^b^ Values are given as means, lowercase and uppercase letters indicate significant differences at P ≤0.05 levels in the same line (LSD test) comparisons among different treatments and between two cultivation models, respectively. ns denotes no significant different

*significant different at the 0.05 probability level; CM is the cultivation models, R is the rainfall simulator, DI is the deficit irrigation.

### Malondialdehyde (MDA) and proline contents

Under the TF system, MDA and proline contents of wheat flag leaves at different growth stages were significantly greater than that of the RF cultivation system treated with different precipitation and deficit irrigation levels (Tables 4 and 5). Under both cultivation methods, the MDA and proline contents of flag leaves significantly decreased with increasing precipitation and irrigation concentrations at the jointing, flowering, grain filling and maturity stages. The MDA contents under the TF1_150_ treatment at the jointing, flowering, grain filling and maturity stages were significantly greater by 0.15, 0.91, 0.52, and 2.06 μmol g^-1^ FW, respectively, and the proline contents were significantly greater by 0.06, 0.53, 0.43, and 0.38 μmol g^-1^ FW, respectively, than those of the RF1_150_ treatment. The MDA contents under the TF2_150_ treatment, at the jointing, flowering, grain filling and maturity stages were significantly greater by a respective 1.07, 2.87, 1.44, and 3.87 μmol g^-1^ FW, and the proline contents were significantly greater by a respective 0.11, 0.14, 0.36, and 0.29 μmol g^-1^ FW, than those of the RF2_150_ treatment. The MDA contents at the jointing, flowering, grain filling and maturity stages, under the TF3_150_ treatment were significantly greater by 0.42, 2.04, 0.84, and 3.04 μmol g^-1^ FW), respectively, and the proline contents were significantly greater by 0.36, 0.09, 0.12, and 0.22 μmol g^-1^ FW, respectively, than those of the RF3_150_ treatment.

**Table 4:** Malondialdehyde (MDA) content (μmol g^-1^ FW) in winter wheat leaves of different treatments^a^ during 2015–2016 and 2016–2017^b^.

Treatments	Deficit irrigation	MDA content (μmol g^-1^ FW)							
		2015–2016				2016–2017			
		JS	FS	GFS	MS	JS	FS	GFS	MS
RF1	150	4.80e	5.20d	5.09e	8.20e	5.10f	5.69e	5.49f	9.10e
RF1	75	7.09c	9.29b	7.49c	12.29b	7.39c	9.94c	7.89c	13.19c
RF2	150	5.06d	7.09c	5.46e	10.09d	5.36e	7.69d	6.06e	11.39d
RF2	75	8.30b	9.52b	8.70b	12.52b	8.60b	10.12b	9.30b	13.82b
RF3	150	5.65d	9.01b	6.05d	12.01c	5.95d	9.81c	6.75d	13.61b
RF3	75	9.90a	14.37a	10.30a	17.37a	10.20a	15.17a	11.00a	18.97a
	**mean**	**6.80B**	**9.08B**	**7.18B**	**12.08B**	**7.10B**	**9.74B**	**7.75B**	**13.35B**
TF1	150	5.20e	7.11e	5.90f	11.11e	5.56e	7.71f	6.50e	12.41e
TF1	75	7.21c	10.15c	7.91c	14.15c	7.57c	10.75d	8.51c	14.45c
TF2	150	5.55d	8.21d	6.25d	12.21d	5.91e	9.11e	7.05d	13.81d
TF2	75	9.34b	12.23b	10.04b	16.23b	9.70b	13.13b	10.84b	16.83b
TF3	150	5.97d	10.56c	6.67d	14.56c	6.33d	11.56c	7.67d	16.36b
TF3	75	10.2a	16.32a	10.99a	20.32a	10.65a	17.32a	11.99a	21.95a
	**mean**	**7.25A**	**10.76A**	**7.96A**	**14.76A**	**7.62A**	**11.60A**	**8.76A**	**15.97A**
Analysis of variance	CM	*	*	*	*	*	*	*	*
	R	*	*	*	*	*	*	*	*
	DI	*	*	*	*	*	*	*	*
	CM x R	*	ns	*	*	ns	*	ns	ns
	CM x DI	ns	ns	ns	ns	*	ns	ns	ns
	R x DI	ns	ns	ns	ns	ns	ns	ns	ns
	CM x R x DI	ns	ns	ns	ns	ns	ns	ns	ns

^a^ RF1: ridges covered with plastic film mulch and 275 mm simulated rainfall; RF2: ridges covered with plastic film mulch and 200 mm simulated rainfall; RF3: ridges covered with plastic film mulch and 125 mm simulated rainfall; TF1: traditional flat cultivation and 275 mm simulated rainfall; TF2: traditional flat cultivation and 200 mm simulated rainfall; TF3: traditional flat cultivation and 125 mm simulated rainfall; _150_: 150 mm deficit irrigation; _75_: 75 mm deficit irrigation. Abbreviations are: JS: jointing stage, FS: flowering stage, GFS: grain filling stage, MS: maturity stage.

^b^ Values are given as means, lowercase and uppercase letters indicate significant differences at P ≤0.05 levels in the same line (LSD test) comparisons among different treatments and between two cultivation models, respectively. ns denotes no significant different

*significant different at the 0.05 probability level; CM is the cultivation models, R is the rainfall simulator, DI is the deficit irrigation.

**Table 5:** Proline content (μmol g^-1^ FW) in winter wheat leaves of different treatments^a^ during 2015–2016 and 2016–2017^b^.

Treatments	Deficit irrigation	Proline content (μmol g^-1^ FW)							
		2015–2016				2016–2017			
		JS	FS	GFS	MS	JS	FS	GFS	MS
RF1	150	0.11d	0.38e	0.18e	0.17d	0.13d	0.41f	0.20e	0.18d
RF1	75	0.13d	0.49d	0.30d	0.25d	0.15d	0.54e	0.32d	0.26d
RF2	150	0.17c	0.81c	0.26d	0.24d	0.20c	0.83d	0.30d	0.22d
RF2	75	0.21c	1.89b	0.64c	0.60c	0.26c	1.94c	0.67c	0.58c
RF3	150	0.39b	1.81b	0.97b	0.77b	0.43b	1.80b	0.99b	0.71b
RF3	75	0.50a	3.03a	2.91a	1.03a	0.59a	2.89a	2.83a	0.97a
	**mean**	**0.25B**	**1.40B**	**0.88B**	**0.51B**	**0.29B**	**1.40B**	**0.89B**	**0.49B**
TF1	150	0.16e	0.66f	0.50d	0.48d	0.17d	0.69e	0.52e	0.49d
TF1	75	0.19d	1.01d	0.74c	0.63c	0.22d	1.06c	0.76d	0.64c
TF2	150	0.24d	0.94e	0.81c	0.70c	0.27d	0.96d	0.84d	0.68c
TF2	75	0.32c	2.03c	1.01b	0.89b	0.34c	2.08b	0.99c	0.87b
TF3	150	0.48b	3.03a	1.18b	0.88b	0.51b	3.00a	1.50b	0.82b
TF3	75	0.86a	3.12a	3.03a	1.25a	0.90a	3.09a	2.99a	1.19a
	**mean**	**0.38A**	**1.80A**	**1.21A**	**0.81A**	**0.40A**	**1.81A**	**1.27A**	**0.78A**
Analysis of variance	CM	*	*	*	*	*	*	*	*
	R	*	*	*	*	*	*	*	*
	DI	*	*	*	*	*	*	*	*
	CM x R	ns	*	ns	ns	ns	*	ns	ns
	CM x DI	ns	ns	ns	ns	ns	ns	ns	ns
	R x DI	ns	ns	ns	ns	ns	ns	ns	ns
	CM x R x DI	ns	ns	ns	ns	ns	ns	ns	ns

^a^ RF1: ridges covered with plastic film mulch and 275 mm simulated rainfall; RF2: ridges covered with plastic film mulch and 200 mm simulated rainfall; RF3: ridges covered with plastic film mulch and 125 mm simulated rainfall; TF1: traditional flat cultivation and 275 mm simulated rainfall; TF2: traditional flat cultivation and 200 mm simulated rainfall; TF3: traditional flat cultivation and 125 mm simulated rainfall; _150_: 150 mm deficit irrigation; _75_: 75 mm deficit irrigation. Abbreviations are: JS: jointing stage, FS: flowering stage, GFS: grain filling stage, MS: maturity stage.

^b^ Values are given as means, lowercase and uppercase letters indicate significant differences at P ≤0.05 levels in the same line (LSD test) comparisons among different treatments and between two cultivation models, respectively. ns denotes no significant different

*significant different at the 0.05 probability level; CM is the cultivation models, R is the rainfall simulator, DI is the deficit irrigation.

### H_2_O_2_ and O_2_^-^ contents

**Fig 6:**
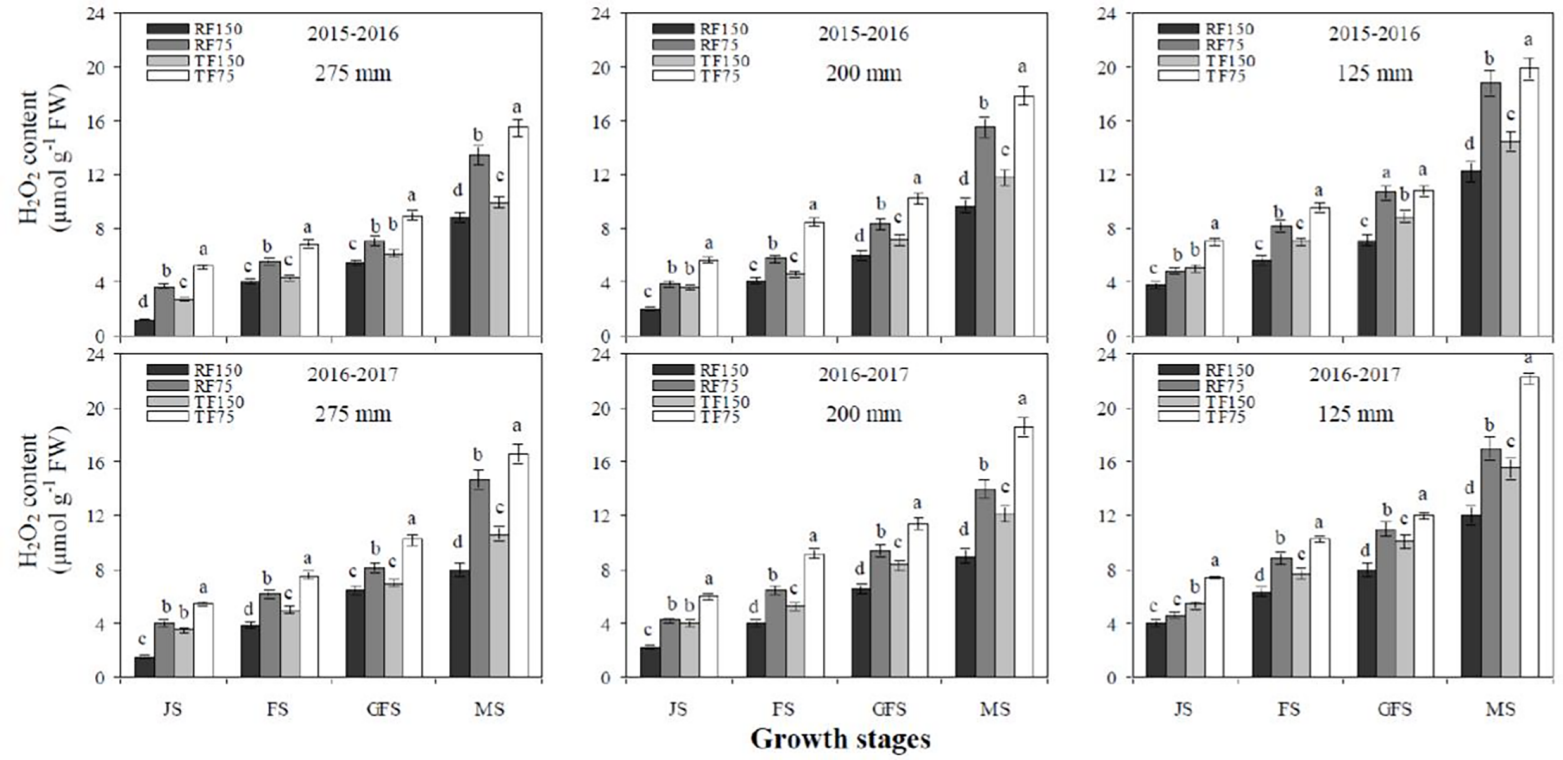
Effects of different cultivation models and deficit irrigation on hydrogen peroxide (H_2_O_2_) content of winter wheat flag leaves under simulated rainfall conditions during 2015–2017 growing seasons. Note: RF1: ridges covered with plastic film mulch and 275 mm simulated rainfall; RF2: ridges covered with plastic film mulch and 200 mm simulated rainfall; RF3: ridges covered with plastic film mulch and 125 mm simulated rainfall; TF1: traditional flat planting and 275 mm simulated rainfall; TF2: traditional flat planting and 200 mm simulated rainfall; TF3: traditional flat planting and 125 mm simulated rainfall; 150: 150 mm deficit irrigation; 75: 75 mm deficit irrigation. Bars represent the LSD at p = 0.05 (n = 3). Abbreviations are: JS: jointing stage, FS: flowering stage, GFS: grain filling stage, MS: maturity stage.

**Fig 7:**
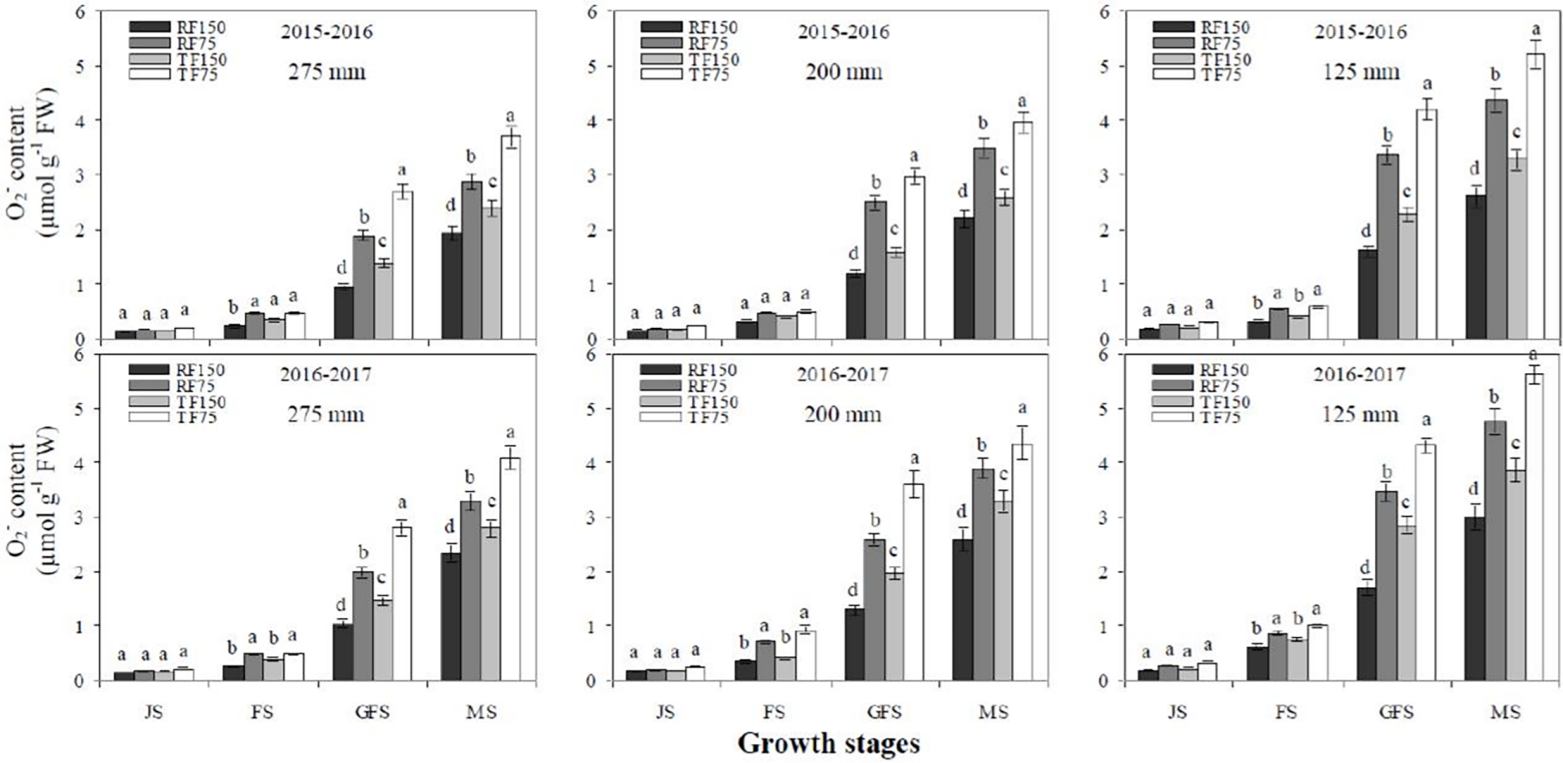
Effects of different cultivation models and deficit irrigation on superoxide radicals (O_2_^-^) content of winter wheat flag leaves under simulated rainfall conditions during 2015–2017 growing seasons. Note: RF1: ridges covered with plastic film mulch and 275 mm simulated rainfall; RF2: ridges covered with plastic film mulch and 200 mm simulated rainfall; RF3: ridges covered with plastic film mulch and 125 mm simulated rainfall; TF1: traditional flat planting and 275 mm simulated rainfall; TF2: traditional flat planting and 200 mm simulated rainfall; TF3: traditional flat planting and 125 mm simulated rainfall; 150: 150 mm deficit irrigation; 75: 75 mm deficit irrigation. Bars represent the LSD at p = 0.05 (n = 3). Abbreviations are: JS: jointing stage, FS: flowering stage, GFS: grain filling stage, MS: maturity stage.

Under the TF cultivation method, the H_2_O_2_ and O_2_^-^ contents of flag leaves significantly increased at each deficit irrigation and simulated rainfall levels than in the RF system (Figs 6 and 7). The H_2_O_2_ and O_2_^-^ contents of flag leaves increased gradually at the jointing to flowering stage and from the flowering to grain filling stages. Moreover, H_2_O_2_ and O_2_^-^ concentrations rapidly increased from the grain filling to maturity stages under both cultivation models at each deficit irrigation and simulated rainfall levels. However, the two years of data from the four different growth stages of winter wheat revealed that the TF1_150_, TF2_150_ and TF3_150_ treatments had produced, on average, significantly more (6.14, 7.10, 11.01 μmol g^-1^ FW) H_2_O_2_ and (1.13, 1.25, 1.62 μmol g^-1^ FW) O_2_^-^ in the flag leaves than the leaves of the RF system. The H_2_O_2_ and O_2_^-^ contents of flag leaves were significantly higher under the TF3_75_ treatment, compared with all other treatments at the jointing, flowering, grain filling and maturity stages. During the maturity stage, H_2_O_2_ and O_2_^-^ contents of flag leaves reached the highest values under the TF cultivation method at each precipitation and deficit irrigation level that were also greater than those of the RF system.

### ET, WUE and winter wheat production

There were clear differences in ET rate among the various treatments of wheat crop, but the trend of ET rate in each year was similar (Table 6). The RF system under deficit irrigation with simulated precipitation significantly (P< 0.05) reduces the ET rate than that of TF planting in each study year. The average of two years data indicated that under the RF cultivation model, enhanced soil water and decreased soil evaporation; as a result significantly reduced ET rate by 35.8%, in 2015–16, and 26.9% in 20116–17 study year, then that of TF planting, respectively. The mean data of two years showed that ET rate of RF1_150_ and RF1_75_ treatments were significantly decreased by 28.1% and 27.8%, than that of TF1_150_ and TF1_75_ treatments, respectively. The mean ET rate of RF2_150_, and RF2_75_ treatments were significantly declined by 32.7% and 33.7%, compared with TF2_150_ and TF2_75_ treatments, respectively. The ET rate was significantly declined by 31.7% in RF3_150_, and 37.3% in RF3_75_ treatment, then that of TF3_150_ and TF3_75_ treatments, respectively.

**Table 6:** Effects of different treatments^a^ on grain yield (t ha^-1^), evapotranspiration (ET, mm) and water use efficiency (WUE, kg ha^-1^ mm^-1^) of winter wheat during 2015–2016 and 2016–2017 growing season^b^.

Treatments	Deficit irrigation	Grain yield (t ha^-1^)		ET (mm)		WUE (kg ha^-1^ mm^-1^)	
		2015–2016	2016–2017	2015–2016	2016–2017	2015–2016	2016–2017
RF1	150	7.53±0.19a	7.79±0.18a	323.92±36.3a	332.43±38.4a	23.26±0.8b	23.43±0.7b
RF1	75	7.07±0.58b	6.62±0.68b	314.22±41.4a	325.55±39.3a	22.51±0.6b	20.35±0.9c
RF2	150	7.40±0.58a	7.57±0.64a	260.62±37.4b	260.36±41.5b	28.39±1.0a	29.06±1.2a
RF2	75	6.53±0.51b	5.99±0.52b	259.60±43.2b	250.26±34.3b	25.17±1.1b	23.95±0.9b
RF3	150	5.42±0.38c	3.94±0.41c	241.00±32.1c	249.63±38.5b	22.50±0.8b	15.79±0.8d
RF3	75	4.15±0.52d	2.92±0.50d	215.16±39.8d	221.42±33.2c	19.30±0.7c	13.19±0.6d
	mean	6.35A	5.81A	269.09B	273.28B	23.52A	20.96A
TF1	150	6.86±0.46a	6.99±0.59a	482.32±45.3a	430.05±42.2a	14.22±1.2a	16.24±0.9a
TF1	75	6.42±0.18a	5.74±0.15b	463.05±39.6a	423.42±37.6a	13.85±0.9b	13.55±0.7b
TF2	150	6.26±0.32a	6.33±0.22a	414.52±35.2b	359.84±34.2b	15.10±1.1a	17.58±1.1a
TF2	75	5.62±0.31b	4.60±0.23c	410.91±41.6b	357.99±40.1b	13.68±0.6b	12.86±1.2b
TF3	150	4.47±0.37c	3.72±0.35d	376.48±43.8c	341.50±46.1c	11.88±0.8c	10.88±0.6b
TF3	75	3.80±0.26d	2.69±0.23e	367.05±37.5c	329.01±38.2c	10.36±0.5c	8.17±0.7c
	mean	5.56B	5.01B	419.06A	373.64A	13.18B	13.21B
Analysis of variance	CM	*	*	*	*	*	*
	R	*	*	*	ns	*	*
	DI	ns	ns	ns	ns	ns	ns
	CM x R	*	ns	*	*	*	*
	CM x DI	ns	ns	ns	ns	ns	*
	R x DI	ns	*	ns	ns	ns	ns
	CM x R x DI	*	*	ns	ns	*	*

^a^ RF1: ridges covered with plastic film mulch and 275 mm simulated rainfall; RF2: ridges covered with plastic film mulch and 200 mm simulated rainfall; RF3: ridges covered with plastic film mulch and 125 mm simulated rainfall; TF1: traditional flat cultivation and 275 mm simulated rainfall; TF2: traditional flat cultivation and 200 mm simulated rainfall; TF3: traditional flat cultivation and 125 mm simulated rainfall; _150_: 150 mm deficit irrigation; _75_: 75 mm deficit irrigation.

^b^ Values are given as means±SD, lowercase and uppercase letters indicate significant differences at P ≤0.05 levels in the same line (LSD test) comparisons among different treatments and between two cultivation models, respectively. ns denotes no significant different

*significant different at the 0.05 probability level; CM is the cultivation models, R is the rainfall simulator, DI is the deficit irrigation.

The WUE and grain yield were significantly improved by interactive effect of cultivation models with deficit irrigation and simulated rainfall levels in both study years (Table 6). The WUE and grain yield significantly enhanced with increasing supplemental irrigation and rainfall levels under both cultivation models, but differences were not significant when the precipitation was more them 200 mm. The WUE improved with the increasing of grain yield. The average of two years data indicated that WUE under the RF cultivation model significantly improved by 58.6% in 2015–16 year and 78.4% in 2016–17 year, then that of the TF planting model, respectively. The mean WUE of two study years indicated that under RF1_150_ and RF1_75_ treatments were significantly improved by 53.3% and 56.4%, then that of TF1_150_ and TF1_75_ treatments. The WUE of RF2_150_ and RF2_75_ treatments were significantly improved by 75.8% and 85.1%, then that of TF2_150_ and TF2_75_ treatments. When compared with TF3_150_ and TF3_55_ treatments, the mean WUE was significantly improved by 68.3% in RF3_150_ treatment and 75.3% in RF3_75_ treatment, respectively. The grain yield led to be produced maximum under the RF system with 150 mm supplemental irrigation and 200 mm rainfall concentrations compared with TF planting. The average grain yield of two years revealed that under RF system enhanced soil water and declined ET rate at field scale; as a result, RF system significantly produced maximum 0.79 t ha^-1^ grain yield compared with TF planting. When compared to TF2_150_ and TF2_75_ treatments, the average grain yield of two-year data revealed that RF2_150_ and RF2_75_ treatments were significantly improved by 1.19 t ha^-1^ and 1.15 t ha^-1^, respectively.

### Pearson's correlation coefficients

Table 7 displays Pearson’s correlation coefficients of the antioxidant enzyme activities, reactive oxygen species, photosynthetic pigments, protein, proline and MDA contents of winter wheat flag laves. Significant positive correlations were observed between Pn and SOD, POD, CAT, and APX activities, as well as soluble protein content. Moreover, significant negative correlations were observed between Pn, SOD, POD, CAT, APX, soluble protein and the activities of H_2_O_2_, O_2_, MDA and proline content. The correlation analysis also confirmed significant positive correlations among H_2_O_2_, O_2_, MDA and proline content.

**Table 7:** Pearson's correlation coefficients of antioxidant enzyme activities, reactive oxygen species (ROS), protein, proline and MDA contents of winter wheat flag laves.

	Pn	SOD	POD	CAT	SP	H_2_O_2_	O_2_	PC	MDA	APX
SOD	0.938*	-								
POD	0.951*	0.931*	-							
CAT	0.928*	0.934*	0.991*	-						
SP	0.943*	0.969*	0.949*	0.953*	-					
H_2_O_2_	-0.931*	-0.924*	-0.900*	-0.904*	-0.897*	-				
O_2_	-0.903*	-0.893*	-0.853*	-0.859*	-0.885*	0.981*	-			
PC	-0.959*	-0.851*	-0.865*	-0.829*	-0.866*	0.856*	0.853*	-		
MDA	-0.923*	-0.906*	-0.866*	-0.862*	-0.881*	0.977*	0.985*	0.878*	-	
APX	0.978*	0.958*	0.938*	0.926*	0.963*	-0.928*	-0.900*	-0.906*	-0.899*	-

Pn, net photosynthesis rate; SOD, Superoxide dismutase; POD, peroxidase; CAT, catalase; SP, soluble protein; H_2_O_2_, hydrogen peroxide; O_2_, superoxide radicals; PC, proline content; MDA, malondialdehyde; APX, ascorbate peroxidise.

* Significant at the 0.01 probability level (n = 12).

## Discussion

Farming systems in semi-arid regions of China are significantly dependent upon the amount and distribution of precipitation because they have significant effects on photosynthetic capacity and thus wheat production [38, 27]. Frequently, uneven distribution of rainfall leads to soil drought which negatively affects the photosynthetic capacity and anti-oxidative enzyme activities in the flag leaves of winter wheat and results in drought-induced plant stress during the critical growth stages [7]. Previous studies have reported that the RF cultivation method increased soil water content and storage which significantly improved the anti-oxidative defence system of wheat flag leaves [39, 7]. This study confirmed that the RF system can significantly increase SWC by 30.4% more than that of the TF planting model. The amount in SWC significantly increased from the jointing to the flowering stages, and in contrast, decreased from the flowering to the grain filling and harvesting stages among all the treatments.

The SP content was closely correlated to Pn and significantly improved the photosynthetic capacity of flag leaves. We suspect that greater SP content in flag leaves sustains a higher Pn (Figs 2 and 3). Under drought stress, the sucrose content decreased due to low SP content, which influenced grain filling rate and Pn value [10]. Water stress encourages stomatal closure, which affects the diffusion of CO_2_ from the air to the cell, is the major cause for decrease of Pn value [11, 28]. During drought conditions supplemental irrigation encourages the plant tissue develop, leading to a significant improve the Pn value and SP content of flag leaves [12]. This study also confirmed that the Pn and SP content under the RF1_150_ and RF2_150_ treatments were significantly higher than in the TF1_150_ and TF2_150_ treatments at jointing, flowering and grain filling stages. However, when the precipitation increased from 200 to 275 mm, there was no significant difference recorded for the Pn and SP content under both cultivation methods at different growth stages. A previous study also observed that Pn and SP content of flag leaves in wheat were greater due to the RF cultivation model with plastic film when compared to that of the TF planting without plastic film [40]. Furthermore, we observed that the RF cultivation model had significantly affected the Pn and SP content of flag leaves at each simulated precipitation and deficit irrigation concentrations during later growth stages of winter wheat. At grain filling stage the Pn value and total Chl. ab decrease due to start leaves senesce and the photosynthetic machine quickly disassemble and declines the photosynthetic capacity of flag leaves [13, 14].

Water shortage is always linked with increased oxidative stress owing to enhanced accumulation of ROS [16, 17]. In our study, we determined that H_2_O_2_ and O_2_^-^ contents of flag leaves increased gradually at the jointing to flowering stage and from flowering to grain filling stage, while rapidly increased from grain filling to maturity stage under both cultivation models at each deficit irrigation and simulated rainfall levels. During maturity stage the H_2_O_2_ and O_2_^-^ contents of flag leaves reached to the highest values under the TF cultivation model at each precipitation and deficit irrigation level, than in the RF system. The highest contents of ROS in response to reduced soil water storage [17]. Under severe drought significant increase of H_2_O_2_ and O_2_^-^ production in wheat flag leaves [19]. In the present study, the H_2_O_2_ and O_2_^-^ contents were significantly higher under the TF3_75_ treatment, compared with all other treatments at the jointing, flowering, grain filling and maturity stages. Normal metabolism can be destroying by ROS through oxidative damage to lipids, proteins and nucleic acids. Therefore, oxidative damage negatively affects plant performance, Pn value and chlorophyll contents [18]. ROS caused damage of membrane and produce MDA content. But, crops have developed anti-oxidant defence systems such as SOD, POD, CAT and APX to reduce the H_2_O_2_ and O_2_^-^ production [20]. In the current study, the maximum SOD, POD and CAT activities of flag leaves were recorded under the RF1_150_ treatment at the flowering stage. Subsequently, SOD, POD and CAT activities of flag leaves quickly declined at the maturity stage. Under both cultivation models, SOD, POD and CAT activities of flag leaves significantly increased from the jointing to flowering stage and rapidly declined from the grain filling to maturity stage at each deficit irrigation and simulated rainfall level. [41, 42] Confirmed that with decreasing amounts of irrigation, SP content and SOD, POD and CAT activities of flag leaves in wheat declined, which resulted in an increased rate of leaf senescence during latter growth stages. Similarly, we determined that the RF cultivation methods had significantly higher effects on SOD, POD and CAT activities during later growth stages than the TF cultivation method. Furthermore, there were non-significant effect on the SOD, POD and CAT activities of flag leaves when supply simulated rainfall of 275 mm or 200 mm levels at jointing, flowering, grain filling and maturity stages. The increase of SOD, POD, CAT and APX activities under water stress was also reported by [43]. Thus, increasing SOD, POD, CAT and APX activities and lowering MDA, H_2_O_2_ and O_2_^-^ production in flag leaves may be key strategies of wheat plants to improve their photosynthetic capacity, total Chlcontent and CSI under water stress [44, 21].

MDA content was significantly increased under water stress than that of normal condition and reached to maximum at the mature stage [19, 41]. Our study showed that under both cultivation models, the MDA and proline contents of flag leaves were significantly decreased with increasing precipitation and irrigation amount at jointing, flowering, grain filling and maturity stages. Proline is known to be the first response of plants exposed to water stress and plays a significant role in plant stress tolerance [22, 23]. Proline acts as osmolyte and promotes plant damage repair capability by increasing anti-oxidant activity during drought conditions [24]. We concluded that the RF2_150_ treatment was the better water-saving management strategy because the higher free H_2_O_2_ and O_2_ scavenging capacity and lower level of lipid peroxidation indicate a better anti-oxidation defence system that can effectively protect the photosynthetic apparatus in winter wheat.

## Conclusions

Antioxidant enzyme activities and reactive oxygen species (ROS) of wheat flag leaves varied significantly under different combinations of cultivation methods, deficit irrigation regimes and simulated rainfall concentrations. The RF2_150_ treatment raised average soil water content (%) from soil collected from depths of 0–100 cm during the jointing and flowering stages of wheat, and had the highest net photosynthesis rate (Pn) in the flag leaves. Moreover, such improvements were due to reducing MDA content and oxidative damage during different growth stages of winter wheat flag leaves. The RF2_150_ treatment significantly increased SOD, POD, CAT, APX activities and soluble protein content (SP) in flag leaves at different growth stages of winter wheat and attained the highest values at the flowering stage, whereas, contents of proline, MDA, H_2_O_2_, and O_2_ in flag leaves significantly declined and reached a maximum value at the maturity stage. The higher free ROS scavenging capacity and better antioxidant enzyme activities under the RF2_150_ treatment were due to the lower level of lipid peroxidation, which effectively protected the photosynthetic machinery. These results indicate that the RF2_150_ treatment significantly delayed senescence of leaves, and increased SWC, Pn, WUE, antioxidant enzyme activities and yield of winter wheat.

## Supporting information

S1 Data File Soil water contents. (XLS)

S2 Data File Net photosynthesis rate. (XLS)

S3 Data File Antioxidant enzyme activities, reactive oxygen species (ROS), APX, proline content, soluble protein content, praline content. (XLS)

S4 Data File Grain yield (t ha^-1^), evapotranspiration (ET, mm) and water use efficiency (WUE, kg ha^-1^ mm^-1^). (XLS)

S5 Data File Pearson's correlation coefficients DATA. (XLS)

## References

[1] Shi J., Yasuor H., Yermiyahu U., Zuo Q., Ben-Gal A., 2014: Dynamic responses of wheat to drought and nitrogen stresses during re-watering cycles. Agric. Water Manage. 146, 163–172.

[2] Villagra P. E., Cavagnaro J. B., 2006: Water stress effects on the seedling growth of Prosopis argentina and Prosopis alpataco. J. Arid Environ. 64, 390–400.

[3] Vinocur B., Altman A., 2005: Recent advances in engineering plant tolerance to abiotic stress: achievements and limitations. Curr. Opin. Biotechnol. 16, 123–132. doi: 10.1016/j.copbio.2005.02.001 15831376 10.1016/j.copbio.2005.02.001

[4] Guo Z.J., Yu Z.W., Wang D., Shi Y., Zhang Y.L., 2014a: Photosynthesis and winter wheat yield responses to supplemental irrigation based on measurement of water content in various soil layers. Field Crops Res. 166, 102–111.

[5] Thompson T.L., Pang H.C., Li Y.Y., 2009: The potential contribution of subsurface drip irrigation to water-saving agriculture in the western USA. Agricultural Sciences in China 8, 850–854 (in Chinese).

[6] Tian Y., Su D. R., Li F. M., Li X. L., 2003: Effect of rainwater harvesting with ridge and furrow on yield of potato in semiarid areas. Field Crops Research, 84, 385–391.

[7] Wang Y.J., Xie Z.K., Malhi S.S., Vera C.L., Zhang Y.B., Wang J.N., 2009: Effects of rainfall harvesting and mulching technologies on water use efficiency and crop yield in the semi-arid Loess Plateau, China. Agricultural Water Management, 96, 374–382.

[8] Sawhney V., Singh D.P., 2002: Effect of chemical desiccation at the post-anthesisstage on some physiological and biochemical changes in the flag leaves of con-trasting wheat genotypes. Field Crops Res. 77, 1–6.

[9] Tan W., Liu J., Dai T., Jing Q., Cao W., Jiang D., 2008: Alternations in photosynthesis and antioxidant enzyme activity in winter wheat subjected to post-anthesis water-logging. Photosynthetica, 46, 21–27.

[10] Gupta A.K., Kaur K., Kaur N., 2011: Stem reserve mobilization and sink activity inwheat under drought conditions. Am. J. Plant Sci. 2, 70–77.

[11] Reddy A. R., Chaitanya K. V., Vivekanandan M., 2004: Drought-induced responses of photosynthesis and antioxidant metabolism in higher plants. Journal of plant physiology. 11, PMID: 15602811. 10.1016/j.jplph.2004.01.013 15602811

[12] Cui Y., Tian Z., Zhang X., Muhammad A., Han H., Jiang D., Cao W., Dai T., 2015: Effect of water deficit during vegetative growth periods on post-anthesis photosynthetic capacity and grain yield in winter wheat (Triticum aestivum L.). Acta Physiol. Plant. 37, 196.

[13] Ding R. X., Zhang B. J., Fan H. L., 2004: Effect of the super water-absorbent polymers Kehan 98 on winter wheat physiology characteristic and yield. Triticeae Crops, 5, 110–112. (in Chinese)

[14] Wang Q., Zhang E. H., Li F. M., Li F. R., 2008: Runoff efficiency and the technique of micro-water harvesting with ridges and furrows, for potato production in semi-arid areas. Water Resources Management, 22, 1431–1443.

[15] Irada M.H., 2012: Photosynthetic characteristics and enzymatic antioxidant capacity of leaves from wheat cultivars exposed to drought. Biochimica et Biophysica Acta, 1817, 1516–1523. doi: 10.1016/j.bbabio.2012.02.037 22417798 10.1016/j.bbabio.2012.02.037

[16] Beck E.H., Fetitig S., Knake C., Hartig K., Bhattarai T., 2007: Specific and unspecificresponses of plants to cold and drought stress. J. Biosci. 32, 501–510. 17536169 10.1007/s12038-007-0049-5

[17] Miller G., Suzuki N., Ciftci-Yilmaz S., Mittler R., 2010: Reactive oxygen species homeostasis and signaling during drought and salinity stresses. Plant Cell Environ. 33, 453–467. doi: 10.1111/j.1365-3040.2009.02041.x 19712065 10.1111/j.1365-3040.2009.02041.x

[18] Fazeli F., Ghorbanli M., Niknam V., 2007: Effect of drought on biomass, protein content, lipid peroxidation and antioxidant enzymes in two sesame cultivars. Biol. Plant. 51, 98–103.

[19] Sairam R.K., Srivastava G.C., 2001: Water stress tolerance of wheat (*Triticum aestivum* L.): variations in hydrogen peroxide accumulation and antioxidant activity in tolerant and susceptible genotypes. J. Agron. Crop Sci. 186, 63–70.

[20] Farooq M., Wahid A., Kobayashi N., Fujita D., Basra S.M., 2009: Plant drought stress: effects, mechanisms and management. Agron. Sustainable Dev. 29, 185–212.

[21] Fotelli M.N., Rennenberg H., Gessler A., 2002: Effects of drought on the competitive interference of an early successional species (Rubus fruticosus) on Fagus sylvatica L. seedlings: N-15 uptake and partitioning, responses of amino acids and other N compounds. Plant Biol. 4, 311–320.

[22] Anjum S.A., Wang L.C., Farooq M., Hussain M., Xue L.L. and Zou C.M., 2011: Brassinolide application improves the drought tolerance in maize through modulation of enzymatic antioxidants and leaf gas exchange. J. Agron. Crop Sci. 197, 177–185.

[23] Lum M.S., Hanafi M.M., Rafii Y.M., Akmar A.S.N., 2014: Effect of drought stress on growth, proline and antioxidant enzyme activities of upland rice. J. Anim. Plant Sci. 24, 1487–1493.

[24] Kaur K., Kaur N., Gupta A.K., Singh, 2013: Exploration of the antioxidative defense system to characterize chickpea genotypes showing differential response towards water deficit conditions. Plant Growth Regul. 70, 49–60.

[25] Blandino M., Reyneri A., 2009: Effect of fungicide and foliar fertilizer application to winter wheat at anthesis on flag leaves senescence, grain yield, flour bread-making quality and DON contamination. Eur. J. Agron. 30, 275–282.

[26] Ali S., Xu Y., Ma X., Ahmad I., Kamran M., Dong Z., Cai T., Jia Q., Ren X., Zhang P., and Jia Z., 2017: Planting models and deficit irrigation strategies to improve wheat production and water use efficiency under simulated rainfall conditions. Front. Plant Sci. 8:1408 doi: 10.3389/fpls.2017.01408 28878787 10.3389/fpls.2017.01408 PMC5572266

[27] Lichtenthaler H., Wellburn A.R., 1983: Determination of total carotenoids and chlorophyll a and chlorophyll b leaf extracts in different solvents. Biochem Soc Trans. 603: 591–592.

[28] Sairam R. K., Deshmukh P. S., Shukla D.S., 1997: Tolerance of drought and temperature stress in relation to increased antioxidant enzyme activity in wheat. J Agron Crop Sci. 178, 171–178.

[29] Li H.S., 2000: Principles and Techniques of Plant Physiological Experiment. Higher Education Press, Beijing, pp. 119–120 (in Chinese).

[30] Amalo K., Chen G.X., Asade K., 1994: Separate assays specific for ascorbate peroxidase and guaiacal peroxidase and for the chloroplastic and cytosolic isozymes of ascorbate peroxidase implants. Plant Cell Physiol. 35, 497–504.

[31] Zhang X.Z., 1992 Crop Physiology Research Methods: China Agricultural Press, Beijing, pp. 131–207 (in Chinese).

[32] Read S.M., Northcote D.H., 1981: Minimization of variation in the response to different protein of the Coomassic Blue G dye binding assay for protein. Anal. Biochem.116, 53–64. 7304986 10.1016/0003-2697(81)90321-3

[33] Elstner E.F, Heupel A., 1976: Inhibition of nitrite formation from hydroxyl ammonium chloride: A simple assay for superoxide dismutase. Analytical Biochemistry, 70, 616–620. 817618 10.1016/0003-2697(76)90488-7

[34] Jiang M.Y., Zhang J.H., 2001: Effect of abscisic acid on active oxygen species, antioxidative defence system and oxidative damage in leaves of maize seedlings. Plant Cell Physiol. 42, 1265–1273. 11726712 10.1093/pcp/pce162

[35] Brennan T., Frenkel C., 1977: Involvement of hydrogen peroxide in the regulation of senescence in pear. Plant Physiol. 59, 411–416 16659863 10.1104/pp.59.3.411 PMC542414

[36] Jiang M, Zhang J., 2002: Involvement of plasma-membrane NADPH oxidase in abscisic acid- and water stress-induced antioxidant defense in leaves of maize seedlings. Planta, 215, 1022–1030. doi: 10.1007/s00425-002-0829-y 12355163 10.1007/s00425-002-0829-y

[37] Bates L.S., Waldren R.P., Teare I.D., 1973: Rapid determination of free proline for water-stress studies. Plant Soil, 39, 205–207.

[38] Ren X., Jia Z., Chen X., 2008: Rainfall concentration for increasing corn production under semiarid climate. Agr. Water Manage. 95, 1293–1302.

[39] Noctor G., Foyer C.H., 1998: Ascorbate and glutathione: keeping active oxygen under control. Annu. Rev. Plant Physiol. Plant Mol. Biol. 49, 249–279. doi: 10.1146/annurev.arplant.49.1.249 15012235 10.1146/annurev.arplant.49.1.249

[40] Wu F., Bao W., Li F., Wu N., 2004: Effects of water stress and nitrogen supply on leaf gas exchange and fluorescence parameters of *Sophora davidii* seedlings. Photosynthetica. 1, doi: 10.1007/s11099-008-0008-x

[41] Wang X., Wang L., Shangguan Z., 2016: Leaf Gas Exchange and Fluorescence of Two Winter Wheat Varieties in Response to Drought Stress and Nitrogen Supply. PLoS ONE 11: e0165733 doi: 10.1371/journal.pone.0165733 27802318 10.1371/journal.pone.0165733 PMC5089754

[42] Yu Z.W., Yue S.S., Shen C.G., Zhang W., Yu S.L., 1995: Effect on senescence of flagleaves in winter wheat under high yield low norm irrigation conditions. ActaAgron. Sin. 21, 503–508 (in Chinese).

[43] Quartacci M.F., Pinzino S., gherri C., Navari-Izzo C.L.M.F., 1995: Lipid composition and protein dynamics in thylakoids of two wheat cultivars differently sensitive to drought, Plant Physiol. 108, 191–197. 12228463 10.1104/pp.108.1.191 PMC157320

[44] Sairam R.K., Saxena D.C., 1998: Role of antioxidant systems in wheat genotypes tolerance to water stress. Biol. Plant. 41, 387–394.

